# Robust assessment of the time of emergence of precipitation change in West Africa

**DOI:** 10.1038/s41598-020-63782-2

**Published:** 2020-05-06

**Authors:** Marco Gaetani, Serge Janicot, Mathieu Vrac, Adjoua Moise Famien, Benjamin Sultan

**Affiliations:** 1 Laboratoire Atmosphère Milieux Observations Spatiales LATMOS-IPSL, UMR CNRS 8190, Sorbonne Université, UVSQ, Paris, France; 2 Scuola Universitaria Superiore IUSS, Pavia, Italia; 3 Sorbonne Université, IRD, CNRS, MNHN, Laboratoire d’Océanographie et du Climat: Expérimentations et Approches Numériques, LOCEAN, Paris, France; 4 Laboratoire des Sciences du Climat et l’Environnement (LSCE-IPSL) CNRS/CEA/UVSQ, UMR8212, Université Paris-Saclay, Gif-sur-Yvette, France; 5 Université Félix Houphouët Boigny, LAPAMF-UFR SSMT, Abidjan, Côte d’Ivoire; 6 ESPACE-DEV, Univ Montpellier, IRD, Univ Guyane, Univ Reunion, Univ Antilles, Univ Avignon, Maison de la Télédétection, 500 rue Jean-François Breton, Montpellier, F-34093 France

**Keywords:** Climate and Earth system modelling, Projection and prediction

## Abstract

The time of emergence (TOE) of climate change is defined as the time when a new climate state emerges from a prior one. TOE assessment is particularly relevant in West Africa, a region highly threatened by climate change and urgently needing trustworthy climate predictions. In this paper, the TOE of precipitation change in West Africa is assessed for the first time, by analyzing 6 precipitation metrics (cumulated precipitation, number of wet and very wet days, onset and length of the rainy season) computed from the output of 29 state-of-the-art climate models. In West Sahel, climate conditions characterized by reduced occurrence of wet days are likely to emerge before 2036, leading to the possible emergence of a dryer climate in 2028–2052. In East Sahel, a wetter precipitation regime characterized by increased occurrence of very wet days is likely to emerge before 2054. Results do not provide a clear indication about a possible climate shift in the onset and length of the rainy season. Although uncertainty in climate model future projections still limits the robust determination of TOE locally, this study provides reliable time constraints to the expected climate shift in West Africa at the sub-regional scale, supporting adaptation measures to the future change in the precipitation regime.

## Introduction

Anthropogenic climate change is projected to amplify existing climate-related risks and create news risks for natural and human systems. Therefore, detecting the time of emergence (TOE) of climate change, i.e. the time when a new climate state will emerge from the natural variability, is crucial to anticipate and mitigate climate impacts^1^. In particular, assessing TOE at regional scale is of prominent importance, because reliable prevision would be highly valuable in prioritizing mitigation and adaptation measures in regions projected to be sooner affected by climate change. However, although evidences show that global temperature change has already emerged from natural variability^2^, TOE of climate change at seasonal and local scale is still affected by uncertainty.

Assessing climate change emergence is particularly relevant in West Africa, where populations largely relying on rain fed agriculture are highly threatened by climate change and require trustworthy climate predictions^3^. There is agreement on the fact that thermal metrics in West Africa reached a new climate state already by the end of the 20th century^2^. Conversely, West African precipitation challenges the applicability of the TOE concept. Firstly, it is characterized by large multidecadal variability associated to both natural^4^ and anthropogenic^5^ forcings, so that climate change emergence may not necessarily be associated with the anthropogenic forcing^6^. Moreover, state-of-the-art climate models show a large spread in projecting monsoonal precipitation in the 21st century, not only regarding the amplitude, but also regarding the sign of the change^7^. Therefore, the coexistence of wet and dry trends imposes the assessment of TOE robustness for future precipitation changes.

The TOE concept has been used to assess climate shifts in past and future, for various climate variables, such as temperature^8–10^, precipitation^1,11^, climate extremes^2,12^, and sea level^13^. TOE assessment is affected by uncertainties originating from several sources. When applied to climate models, TOE assessment reflects the differences in model designs and performances, as well as in the choice of the emission scenario^14^. TOE assessment is also affected by methodological choices. Assuming that climate evolution is composed of a low-frequency (LF) component, representing an externally forced climate signal, and a high-frequency (HF) component, representing natural variability, TOE can be assessed by detecting the LF signal exceeding a threshold defined using the HF component. In this case, TOE assessment is affected by the methodology to identify the LF component (e.g., linear trend, running mean, spline, or model ensemble mean) and the choice of the natural variability threshold (e.g., different quantiles of the HF component distribution). In addition, if natural variability is defined as climate variability in a reference period assumed not to be affected by anthropogenic emissions, TOE assessment is also affected by the choice of the reference period. If no assumptions are made on the shape of HF and LF components of climate evolution, TOE can be assessed by simply detecting significant changes in the distribution of climate variables, in comparison with a reference period. In this case, TOE assessment is affected by the choice of the reference period and the methodology used to detect changes in the distribution (e.g., distance measures, statistical hypothesis tests).

In this work, the TOE of precipitation change in West Africa is assessed for the first time. Six precipitation metrics are computed from the output of 29 climate simulations spanning 20th and 21st century (*historical* and *rcp85* CMIP5 experiments respectively^15^, see details in Methods) and three different methodologies for TOE detection are compared: the ‘KS test’ method, which estimates the change in metrics distribution by means of a Kolmogorov-Smirnov test; the ‘smoothing’ and ‘linear trend’ methods, which respectively assume 4th-degree polynomial and linear shapes for the LF component, and the residual standard deviation (STD) as natural variability. The TOE is defined in the period 2006–2009, using the 1950–2005 period as reference. Details on model data, precipitation metrics and TOE detection methods are presented in Methods.

## Results

### Cumulated precipitation

**Figure 1:**
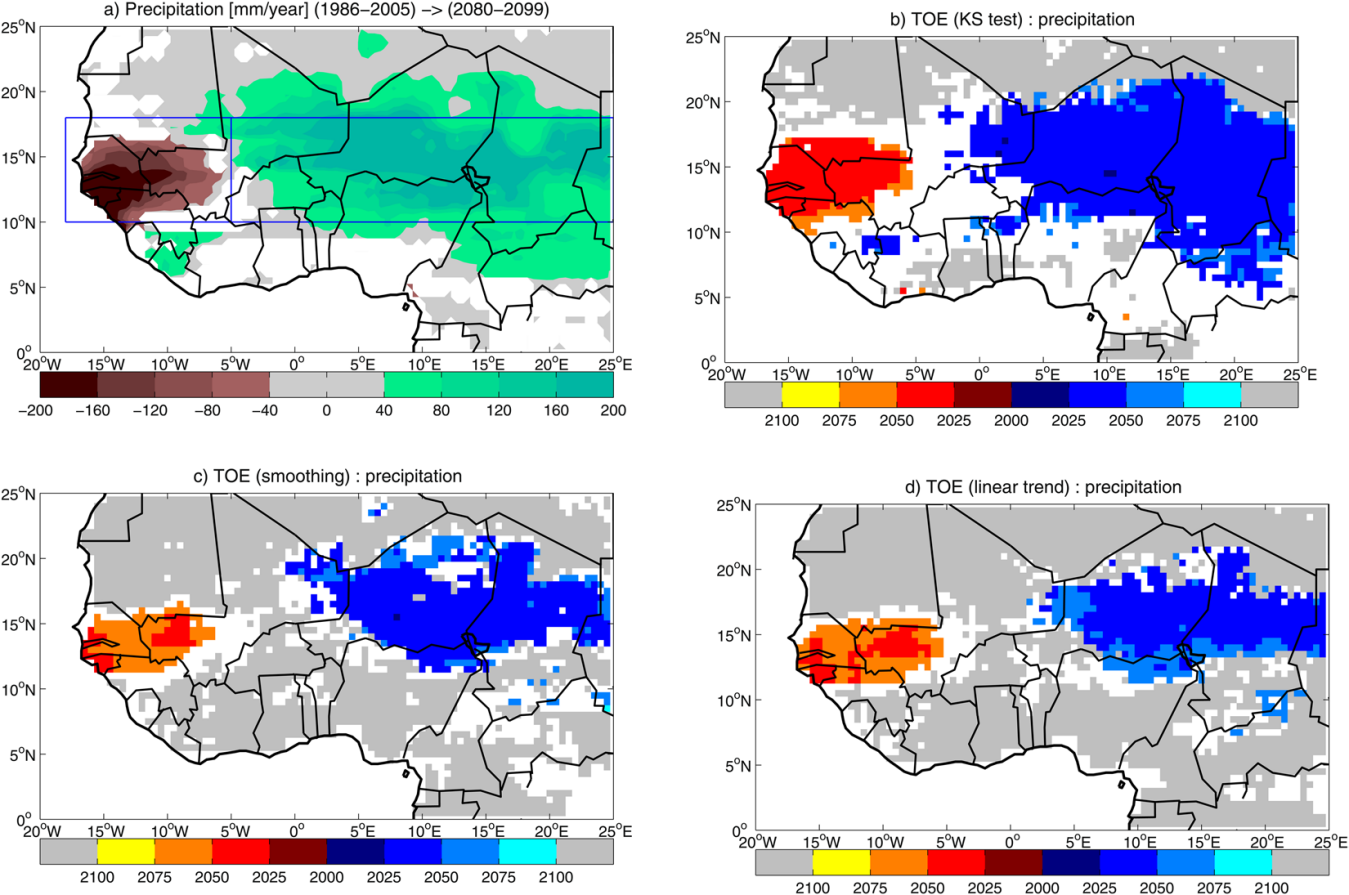
(**a**) Ensemble mean change in July-to-September cumulated precipitation during the 21st century, computed as the difference between 2080–2099 and 1986–2005 averages. Significant values are displayed, after significance is assessed with a Student’s t-test at 95% confidence level. Blue rectangles show the domains where West and East Sahel indices are computed. Multi-model time of emergence (TOE) for cumulated precipitation, estimated by using (**b**) ‘KS test’, (**c**) ‘smoothing’ and (**d**) ‘linear trend’ methods. Blue/red/grey shadings display TOE for positive/negative/no change in cumulated precipitation, based on 50% multi-model agreement. White areas indicate that multi-model TOE cannot be assessed (see Methods Section for details on the assessment of multi-model TOE).

At the end of the 21st century, the ensemble mean (Fig. 1a) shows a change pattern in July-to-September (JAS) cumulated precipitation characterized by a dry-wet dipole in the west-east direction^7^, with deficits in Sahel west of 5°W, and increments east of 5°W. This pattern has been related to the competing effects of the anthropogenic global warming, which stabilizes tropical troposphere and weakens the WAM dynamics^16^, provides moisture advection from North Atlantic to trigger deep convection over West Africa^17^, and intensifies the cyclonic circulation associated with the Saharan heat low^16^, favoring moisture convergence over central-east Sahel^18^. The inter-model spread in projections of cumulated precipitation is characterized by computing regional indices for West and East Sahel (see Fig. 1a and Methods). In West Sahel, future projections simulate 75 mm/year ensemble mean deficit in the period 2080–2099 (13% deficit, Table S1), with 20 out of 29 models (69% of the ensemble) simulating a significant negative change. In East Sahel, future change results in a 135 mm/year ensemble mean increase (+35%, Table S1), with 20 models (69% of the ensemble) simulating a significant positive change. The multi-model agreement on the projection shows that the dry-wet dipole is a robust feature in climate model simulations of future precipitation in the Sahel.

As expected, the spatial pattern of multi-model TOE for cumulated precipitation reproduces the pattern of the 21st century change, showing TOE associated with negative changes in West Sahel and positive changes in East Sahel (Fig. 1b–d). When the ‘KS test’ method is used (Fig. 1b), multi-model TOE shows that climate change emerges in the Sahel west of 5°W and east of 0°E, while Sahara and the central coast of Guinea are not affected. However, in central Sahel and in the most of the coast of Guinea, no information on TOE is provided by the ensemble. When the ‘smoothing’ and ‘linear trend’ methods are used (Fig. 1c,d), the area where TOE is defined is reduced, as well as the no-information area, and the region is dominated by no climate change emergence. However, requesting higher robustness (i.e., 2/3 of ensemble agreement instead of 50%) leads to a deterioration of the TOE assessment in West Africa, where TOE and no-TOE areas shrink or even disappear, and no-TOE-assessment area dominates (Fig. S1).

**Figure 2:**
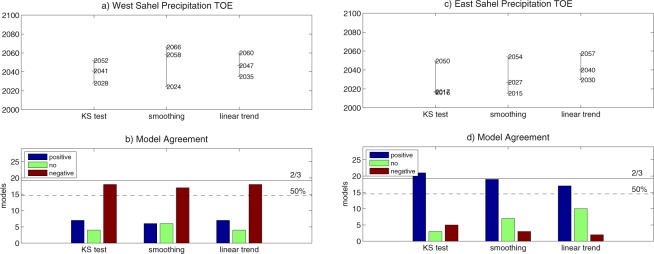
Time of emergence (TOE) for July-to-September cumulated precipitation in (**a,b**) West and (**c,d**) East Sahel estimated by using ‘KS test’, ‘smoothing’ and ‘linear trend’ methods: (**a,c**) multi-model TOE and associated confidence interval (CI); (**b,d**) multi-model agreement (thresholds for 50% and 2/3 of the ensemble are displayed).

Moderate robustness is also revealed by assessing TOE at regional scale instead of pixel-by-pixel. When the regional index for cumulated precipitation in West Sahel is analyzed, the ensemble agrees on the emergence of a negative change, though robustness is only moderate (Fig. 2a,b, Table S2). By using the ‘KS test’ method, 62% multi-model agreement is for TOE in 2041, with 24-year confidence interval (CI). Changing the time window used in the Kolmogorov-Smirnov test to estimate the TOE does not affect the multi-model agreement (see Table S3). However, a narrower window leads to a 10-year delay in the TOE (Table S3). In the ‘smoothing’ method, 59% agreement leads to delayed TOE (2058), with larger CI (42 years). When the ‘linear trend’ method is used, the agreement on a negative change is 62%, and TOE is detected in 2047, with 25-year CI. Larger CI associated with the multi-model TOE in ‘smoothing’ suggests that the inclusion of the multidecadal variability in the LF component may contribute to increase uncertainty in the TOE estimation. In East Sahel, robustness in TOE assessment is also moderate (Fig. 2c,d, Table S2). When the ‘KS test’ method is used, 72% of the ensemble agrees on TOE for a positive change, which occurs early in the 21st century (2017), with 34-year CI. Changing the time window in the ‘KS test’ method has a minor impact on the estimation of multi-model TOE (see Table S3). The ‘smoothing’ method leads to 66% multi-model agreement on TOE for a positive change estimated in 2027 (with 39-year CI), while in the ‘linear trend’ method 59% of the ensemble agrees on TOE for a positive change, which is detected in 2040 (with 28-year CI),

### Wet day occurrence

**Figure 3:**
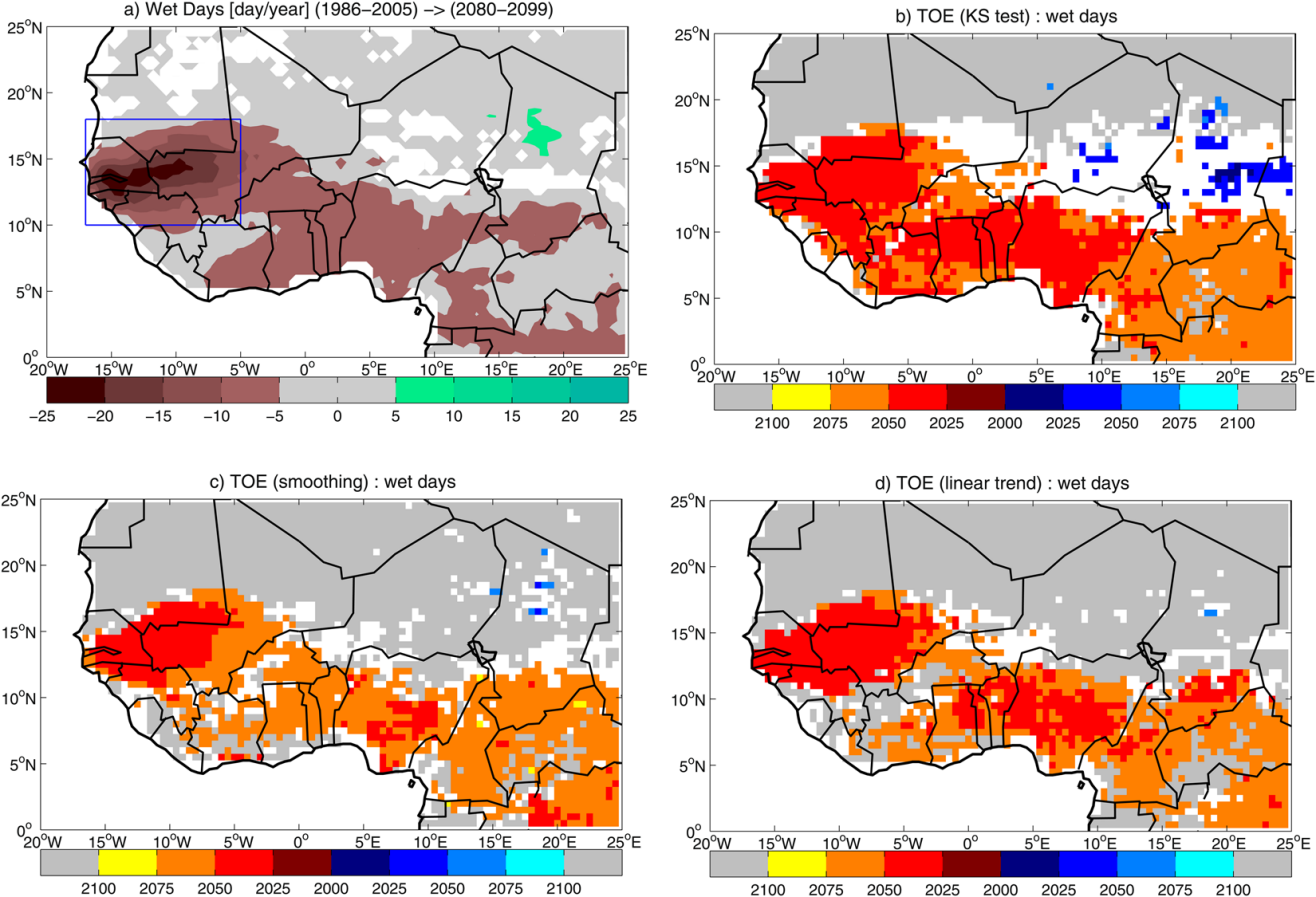
(**a**) Ensemble mean change in the number of wet days in July-to-September during the 21st century, computed as the difference between 2080–2099 and 1986–2005 averages. Significant values are displayed, after significance is assessed with a Student’s t-test at 95% confidence level. The blue rectangle shows the domain where the West Sahel index is computed. Multi-model time of emergence (TOE) for the number of wet days, estimated by using (**b**) ‘KS test’, (**c**) ‘smoothing’ and (**d**) ‘linear trend’ methods. Blue/red/grey shadings display TOE for positive/negative/no change in the number of wet days, based on 50% multi-model agreement. White areas indicate that multi-model TOE cannot be assessed (see Methods Section for details on the assessment of multi-model TOE).

The ensemble mean change in the number of wet days (Fig. 3a) shows a deficit along the Atlantic coast, with a peak in West Sahel. The pattern of change in the number of wet days suggests that the negative trend in total precipitation in West Sahel is related to the deficit in wet days (cf. Fig. 1a). The analysis of the changes in cumulated precipitation associated with wet days shows a pattern almost identical to the change in total cumulated precipitation (Fig. S2a, cf. Fig. 1a), indicating that the reduction in the occurrence of wet days explains the precipitation deficit in West Sahel. Conversely, the simulated increase in wet day precipitation in East Sahel cannot be attributed to the change in the wet day occurrence only (Fig. S2a, cf. Fig. 3a), suggesting that the change in the distribution of precipitation events also plays a role. The West Sahel regional index for the number of wet days shows an ensemble mean of 56.2 wet days for the present-day climatology (Table S1). When the future change is computed, the ensemble shows 11 day/year (−20%) deficit in the multi-model mean (Table S1). Out of the 29 models analyzed, 21 models (72% of the ensemble) simulate a significant negative change, indicating robustness in the projection.

**Figure 4:**
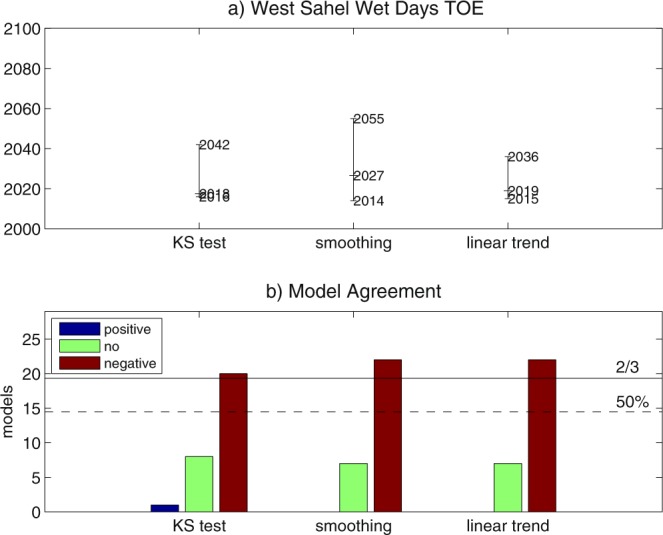
Time of emergence (TOE) for the number of wet days in West Sahel in July-to-September, estimated by using ‘KS test’, ‘smoothing’ and ‘linear trend’ methods: (**a**) multi-model TOE and associated confidence interval (CI); (**b**) multi-model agreement (thresholds for 50% and 2/3 of the ensemble are displayed).

Multi-model TOE for the number of wet days shows spatial patterns very close to expected change at the end of the 21st century (Fig. 3b–d). By using the ‘KS test’ method, TOE for negative changes is detected in West Sahel and along the Coast of Guinea, while no-TOE is detected in the Sahel-Sahara transition zone, and no-TOE-assessment area dominates the most of East Sahel (Fig. 3b). The application of the ‘smoothing’ and ‘linear trend’ methods slightly reduces the area where TOE is detected, increasing the no-TOE area (Fig. 3c,d). Increasing the threshold for multi-model TOE detection to 2/3 of the ensemble limits the homogeneous TOE area to West Sahel, while the most of sub-Saharan West Africa is dominated by no-TOE-assessment locations (Fig. S3). The TOE assessment for the number of wet days averaged in West Sahel shows a robust model response, no matter the method used (Fig. 4, Table S2). Multi-model TOE for a negative change is detected in 69–76% of the ensemble, and is dominated by early 21st century emergence (2018 to 2027), with the ‘smoothing’ method showing the largest CI (41 years). Changing the time window in the ‘KS test’ method leads to slight modifications in the multi-model agreement and the TOE estimation, with minor effects on the TOE assessment (Table S3).

### Very wet day occurrence

**Figure 5:**
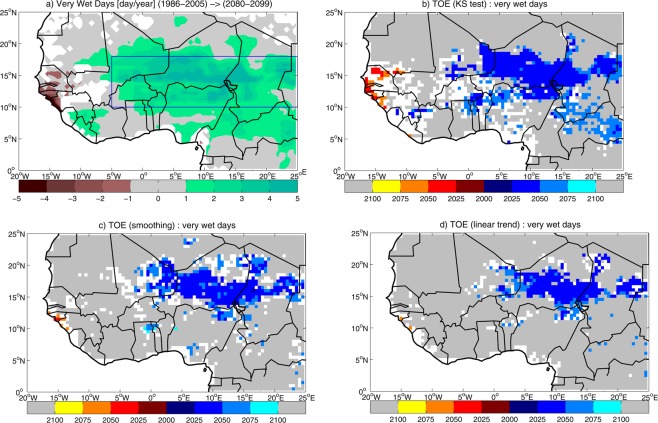
(**a**) Ensemble mean change in the number of very wet days in July-to-September during the 21st century, computed as the difference between 2080–2099 and 1986–2005 averages. Significant values are displayed, after significance is assessed with a Student’s t-test at 95% confidence level. The blue rectangle shows the domain where the East Sahel index is computed. Multi-model TOE for the number of very wet days, estimated by using (**b**) ‘KS test’, (**c**) ‘smoothing’ and (**d**) ‘linear trend’ methods. Blue/red/grey shadings display TOE for positive/negative/no change in the number of very wet days, based on 50% multi-model agreement. White areas indicate that multi-model TOE cannot be assessed (see Methods Section for details on the assessment of multi-model TOE).

The ensemble mean change in the number of very wet days (Fig. 5a) shows an increase in large part of East Sahel. The analysis of cumulated precipitation associated with very wet days shows a precipitation increase in central-eastern West Africa during the 21st century, which accounts for the simulated change in cumulated precipitation in the region (Fig. S2b, cf. Fig. 1a). This suggests that the change in the very wet day regime is at the origin of the projected precipitation change in East Sahel. This result is in line with the observed dramatic increase in the occurrence of extreme storms in the Sahel accompanying the total precipitation recovery in the last decades since the 80s^19^. To explain this intensification, the mechanism invoked is the increased meridional thermal gradient associated with the warming of the Saharan region, which favors the development of intense deep convection in the Sahel. The projected further warming of the Sahara in CMIP5 simulations^20^ is therefore coherent with the increase in the number of very wet days in the 21st century. For present-day climate, the ensemble mean of the number of very wet days averaged in East Sahel is 2.5 day/year, which is doubled in future projections (2.6 day/year increase, corresponding to 103% increase, Table S1). Out of 29 models analyzed, 21 models (72% of the ensemble) simulate significant positive anomalies, indicating robustness in the projection.

**Figure 6:**
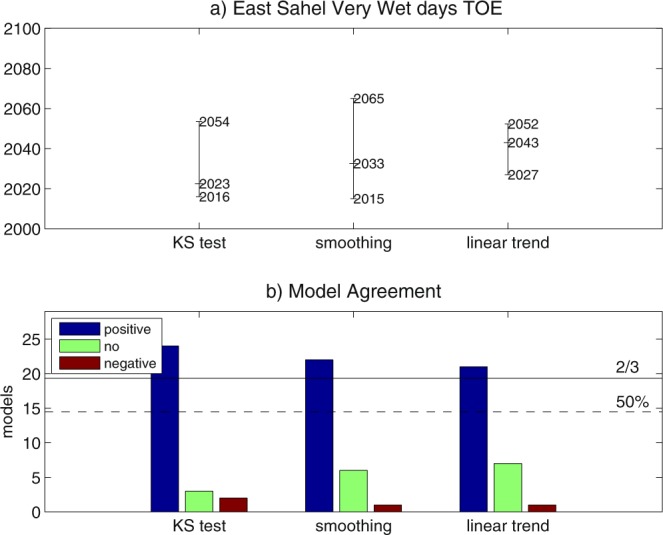
Time of emergence (TOE) for the number of very wet days in East Sahel in July-to-September, estimated by using ‘KS test’, ‘smoothing’ and ‘linear trend’ methods: (**a**) multi-model TOE and associated CI; (**b**) multi-model agreement (thresholds for 50% and 2/3 of the ensemble are displayed).

By using the ‘KS test’ method for the number of very wet days, multi-model TOE for positive changes is detected in East Sahel, with a limited area of multi-model TOE for negative changes in West Sahel and sparse locations of no-TOE-assessment (Fig. 5b). The extension of TOE area in East Sahel slightly shrinks when ‘smoothing’ and ‘linear trend’ methods are used, and no-TOE dominates West Africa (Fig. 5c,d). Increasing the threshold for multi-model TOE detection expands no-TOE-assessment locations across West Africa, reducing TOE and no-TOE areas (Fig. S4). The regionalization of the TOE assessment to East Sahel leads to robust estimation of climate change in the number of very wet days (Fig. 6, Table S2). When ‘linear trend’ and ‘smoothing’ are used, 72% and 76% of the ensemble agrees on TOE for a positive change in 2043 and 2033, with 25 and 50-year CI, respectively. The ‘KS test’ method shows a large 83% ensemble agreement on 2023 as TOE, with 38-year CI. Applying the ‘KS test’ with a narrower (wider) time window leads to a slight delay (anticipation) of the TOE, which does not substantially affect the TOE assessment (Table S3).

### Onset and length of the rainy season

The evolution of the monsoonal season in West Africa is characterized by the meridional migration of the precipitation belt, which crosses the coastline of the Gulf of Guinea in spring, to reach Sahel in full summer, and finally retreat to the coast in September-October^21^. Consistently with observations, the CMIP5 ensemble simulates the onset date of the monsoonal season in April-May in the coastal region and in June in the Sahel (Fig. S5a), while retreat dates range between late September in the Sahel, corresponding to a 3–4 month rainy season, and late October on the coast, corresponding to a 5–6 month season (Fig. S5b,c). At the end of the 21st century, the ensemble mean of the onset date of the WAM shows a generalized delay in the season start (Fig. S6a). The slight delay of the retreat dates (Fig. S6b) does not compensate the late start, so that the season length is reduced (Fig. S6c). The inter-model spread in projections of the onset date is characterized by a computing regional index for West Africa (see Fig. S6 and Methods). In the period 2080–2099, 21 out of 29 models (72% of the ensemble) show a significant delay of the onset date, resulting in an ensemble-mean onset date delayed by 9 days (Table S1). The delay in the onset of the rainy season is accompanied by a slight delay in the retreat of the monsoon (5 days), which results in a 4-day (3%) reduction of the length the rainy season (Table S1). However, this evolution of the rainy season in West Africa does not result in a clear indication of a climate shift during the 21st century (Table S2).

## Discussion and Conclusions

Results show a west-east dipolar pattern for the ensemble mean of the cumulated precipitation at the end of the 21st century in the Sahel. West Sahel is affected by a negative trend, associated with the decrease in the number of wet days, while the positive trend in East Sahel is associated with the increase in the number of very wet days. A delay in the onset date and a shortening in the rainy season is projected in the coastal region and in the Sahel. No matter the method, the assessment of TOE reproduces the spatial pattern of the projected changes in precipitation metrics. Multi-model TOE is defined over larger areas in ‘KS test’ than in ‘smoothing’ and ‘linear trend’, while no-TOE areas are larger in ‘smoothing’ and ‘linear trend’ than in ‘KS test’. Areas of no-TOE-assessment are larger in ‘KS test’ than in ‘smoothing’ and ‘linear trend’. However, TOE assessment based on 2/3 ensemble agreement drastically shrinks TOE and no-TOE areas, leaving West Africa dominated by no-TOE-assessment. Therefore, at local scale a crucial robustness issue emerges in the TOE assessment for WAM precipitation metrics. A further effort is then asked to the climate modelling community to understand the WAM response to global and local drivers, to improve the simulation of its variability and change at time scales from interannual to multidecadal.

Averaging precipitation metrics on regional domains (West and East Sahel) allows a quantitative assessment of TOE robustness. Results show that some features are independent of the used method, and are thus intrinsic to the multi-model simulation of the WAM. Specifically, it emerges that, no matter the assessment method:

- Multi-model TOE determination is robust for the number of wet and very wet days; while it is only moderately robust for cumulated precipitation;
- The climate shift of the WAM cumulated precipitation is associated with the shift in the precipitation regime;
- In West Sahel, multi-model TOE for the occurrence of wet days anticipates the TOE for cumulated precipitation by 23–28 years;
- In East Sahel, multi-model TOE for the occurrence of very wet days is essentially coincident with the TOE for cumulated precipitation.

A method-based analysis also highlights that, for all the indices:

- TOE occurs earlier in ‘KS test’;
- Robustness is the largest when the ‘KS test’ method is used (with the exception of the number of wet days);
- CI is the highest in the ‘smoothing’ method.

The performance of ‘smoothing’ indicates that solving a simple signal-to-noise problem, i.e. decomposing the precipitation metric time series into climate change and natural variability components, leads to reduced agreement and increased uncertainty in multi-model TOE determination. Firstly, this method is based on the exceedance of a threshold, so that TOE detection is dependent on both the magnitude of the trend and the amplitude of natural variability, making less likely the detection of a new climate state. The analysis of individual model TOE detection shows no relationship with the amplitude of natural variability (Fig. S7), while TOE occurrence is mainly associated with the change at the end of 21st century, i.e. the larger (smaller) is the change, the earlier (later) is the TOE (Fig. S8). It is highlighted that the choice of the *rcp85* scenario, which is characterized by the strongest end-of-century radiative forcing in the CMIP5 exercise and produces the most intense climate response, also affects the results. It follows that considering a moderate scenario (e.g. *rcp45*), with proportionally reduced precipitation response, increases the agreement on no-TOE detection (see Fig. S9). Moreover, the correlation between TOE and the end-of-century change is generally lower in ‘smoothing’ than in ‘linear trend’, indicating that including multidecadal variability in the LF component introduces further uncertainty. Indeed, the oscillating nature of the polynomial smoothing of the long term trend may lead to crossings of the threshold not necessarily related to the magnitude of the trend (Fig. S10), increasing the TOE multi-model CI. The large uncertainty shown by the ‘smoothing’ method indicates that multidecadal climate variability may affect the TOE estimation, and its simulation is a key ingredient of climate change predictions in West Africa. Conversely, making no assumptions on the shape of signal and noise leads to reduced uncertainty in the TOE estimation and robust inter-model agreement in ‘KS test’. The method inter-comparison suggests that, in climate model future projections of the WAM dynamics, a possible shift to a new climate state is not only determined by the long term trend, but the interannual and multidecadal variability also play a role. Therefore, improving the simulation of all the components of the climate evolution at different timescales is essential for the reduction of the TOE uncertainty.

It is also highlighted that, in comparison with results based on raw model data, bias correction leads to larger end-of-century differences in precipitation metrics, in general resulting in earlier TOE occurrence, reduced CI and increased model agreement (not shown). It follows that using different bias correction methods might provide slightly different results than those obtained in this study. However, under calibration period, any bias correction technique by construction forces the climate models towards historical patterns of observations to a certain extent, and therefore improves the agreement between models, as seen in this study. Nevertheless, one single bias correction method may not capture the whole uncertainty of the projections. This points out the need for investigating and assessing the potential additional uncertainty associated with the choice of the bias correction method.

This study shows that delivering to policy makers and stakeholders in West Africa reliable and usable information on the change in precipitation regime at the regional scale is possible. By selecting for each index the method showing highest robustness and lowest uncertainty (see Table S2), it is estimated that:

- In West Sahel, a new climate state characterized by reduced wet day occurrence is likely to emerge in 2015–2036, and a regime with reduced cumulated precipitation could emerge in 2028–2052;
- In East Sahel, a new climate state characterized by increased occurrence of very wet days is likely to emerge in 2016–2054, accompanied by a regime with augmented cumulated precipitation likely to emerge in 2016–2050.

The magnitude of the uncertainty associated with the TOE estimation (around 20–40 years) allows the usability of the TOE information to shape adaptation measures in socio-economic sectors taking long time to evolve (e.g. infrastructures, energy production). Conversely, sectors characterized by dynamics at shorter time scales (e.g. agriculture, human health) need a substantial reduction in the TOE uncertainty. Nonetheless, the fact that the lower limits of the multi-model TOE estimation have been already crossed for most of the precipitation metrics highlights the need for urgent implementation of adaptation measures at the regional scale.

## Methods

Precipitation metrics characterizing the monsoonal season are computed for 29 climate models from the CMIP5 archive (Table S4). Model data from *historical* and *rcp85* experiments^15^ have been previously bias corrected, to obtain a homogeneous time series from 1950 to 2099, on a 0.5° regular grid over Africa^22^. Model bias correction has been performed by using the cumulative distribution function-transform (CDF-t) method^23^, which consists in matching the CDF of a simulated climate variable to the CDF of this variable in observations (or references) through a mathematical transformation. The latter is then used to correct biases in *historical* simulations as well as in *rcp85* simulations in accounting for climate change^24^. The CDF-t method is applied to the complete precipitation distribution, namely to zero and non-zero precipitation values. The observation-based reference dataset is WFDEI, the WATCH Forcing Data methodology applied to ERA-Interim data^25^, for the period from 1 January 1979 to 31 December 2013. The WFDEI dataset is a combination of the ERA-Interim daily reanalysis of the European Centre for Medium-Range Weather Forecasts (ECMWF), available at 0.5° resolution, and the observed monthly climate data from the Climate Research Unit (CRU) TS2.1 dataset at 0.5° resolution. WFDEI data are available on a 0.5° regular grid over land, and precipitation in the grid box is defined as the areal average in the box. For an easier comparison with observation, the “raw” simulation data have been interpolated on the 0.5° regular grid of WFDEI using a nearest neighbor approach^22^.

In this study, total cumulated precipitation, number of wet (precipitation in the 1–20 mm/day range) and very wet (precipitation above 20 mm/day) days for the period July-to-September (JAS) are analyzed. In addition, the local onset and retreat dates of the rainy season are determined, by taking into account the combined effect of duration and intensity of rainy sequences^26^. For each year, the daily rainfall accumulated anomaly (A) is computed from 1st January as:

$$A(day)=\mathop{\sum }\limits_{n=1}^{day}r(n)-R,$$

where *r(n)* is the daily precipitation and *R* is the local climatological mean, computed as the mean over the period 1950–2005 and 2006–2099, respectively for the *historical* and *rcp85* experiments (where the climatological mean is lower than 1 mm/day, *R* is set at 1 mm/day). The onset date is defined as the minimum in the rainfall accumulated anomaly time series, i.e., as the first rainy event after a dry sequence, followed by the installation of a wet spell. Following the same principle, the retreat date is defined as the maximum of the accumulated time series. The length of the rainy season is simply defined as the retreat minus onset difference. Compared to methods based on the computation of regional precipitation indices to detect the abrupt northward shift of the rain belt form the coastal region to the Sahel^21^, which only allow the estimation of the onset of the Sahelian phase of the WAM at regional scale, this method makes possible the estimation of the rainy season timing at the local scale across West Africa.

Precipitation metrics are spatially averaged to compute regional indices. West [18°W, 5°W; 10°N, 18°N] and East [5°W-25°E, 10°N-18°N] Sahel indices are defined for cumulated precipitation, and number of wet days and very wet days, by averaging the metrics in areas where the ensemble mean of cumulated precipitation shows homogeneous changes at the end of the 21st century (see Fig. 1a). A West African index [15°W-10°E; 4°N-14°N] is defined for onset and retreat dates and the length of the rainy season by averaging the metrics in an area where the ensemble mean of the onset date shows homogeneous changes at the end of the 21st century (see Fig. S6).

TOE assessment is based on the solution of a signal-to-noise problem, assuming that the externally forced component of the climate variability is the signal and natural variability is the noise. In this study, TOE is assessed by defining the climate change signal in the period 2006–2099, and natural variability in the reference period 1950–2005. Climate change and reference periods are chosen to separate the climate response to a future emission scenario (data from the *rcp85* experiment) from the climate response to observed natural and anthropic forcings during the 20th century (data from the *historical* experiment).

Three different methods are compared:

- In the first method, no assumptions are made on natural variability nor on the forced climate signal. The distribution of a climate variable in a 21-year window sliding in the period 2006–2099 is compared with the distribution of the climate variable in the reference period: TOE is when the first 21-year sliding window distribution is significantly different from the reference period distribution, after a Kolmogorov-Smirnov test at 95% level of confidence^2^. TOE is defined as the middle year in the 21-year window, so that the first possible detection is 2016 and 2089 is the last. The sensitivity of TOE detection to different time windows, namely 15-year and 25-year, is also assessed. This method is referred as ‘KS test’.
- In the second method, the climate variable is decomposed into LF and HF components, separately in the reference and in the 2006–2099 period. The LF component is defined by using a 4th-degree polynomial smoothing of the time series, and residuals represent the HF component. The externally forced climate signal is defined as the LF component in the 2006–2099 period, and natural variability is defined as the standard deviation (STD) of the residuals in the reference period. TOE is defined as the first year for which the LF component exceeds the natural variability^9^. This method is referred as ‘smoothing’. In this case, the smoothed fit of the LF component includes long term trend and multidecadal variability, and natural variability is limited to the interannual variability. However, a considerable part of the WAM multidecadal variability has been attributed to natural climate variability^4^.
- Therefore, a third method is tested to assess the importance of the multidecadal component in the natural variability. In this case, the decomposition is performed by defining the LF component as a linear trend in the climate variable time series, and the HF component as the residuals. TOE is detected as in the ‘smoothing’ method. This method is referred as ‘linear trend’.

TOE is defined, for each model at each grid point, when the emergence is permanent, i.e. only if the new climate state persists until the end of the time series (in 2099). However, a new climate state may be characterized by positive as well as negative trend. Therefore, robust multi-model TOE is defined as the TOE median computed for the models agreeing on the sign of the trend, when the agreement is at least 50% of the ensemble (i.e. 15 out of 29 model ensemble). It follows that, if 50% of the ensemble agrees on no climate change emergence, no multi-model TOE is defined (no-TOE). It is also highlighted that, if there is no 50% agreement on any of the above possibilities, multi-model TOE assessment is not possible, i.e., the model ensemble is not able to provide information on climate change (no-TOE-assessment). Multi-model median is computed following the “one-model-one-vote” approach^27^, i.e. the same weight is given to each model, even if several simulations are performed using different versions of the same model (Table S4). The confidence interval (CI) for the multi-model TOE is defined as the inter-quartile (25th-to-75th percentile) dispersion of individual model TOE.

## Supplementary information

Supplementary information.
