# Supplementary material for: Robust assessment of the time of emergence of precipitation change in West Africa
Source: Sci Rep. 2020 May 6;10:7670. doi: 10.1038/s41598-020-63782-2 (PMC7203108; doi:10.1038/s41598-020-63782-2)
Supplement: Supplementary file 1 — Supplementary information. [file 41598_2020_63782_MOESM1_ESM.pdf]

Robust assessment of the time of emergence of precipitation change in West Africa

Marco Gaetani[1,2,*], Serge Janicot[3], Mathieu Vrac[4], Adjoua Moise Famien[3,5], Benjamin Sultan[6]

[1] LATMOS/IPSL, Sorbonne Université, CNRS, UVSQ, Paris, France
[2] Scuola Universitaria Superiore IUSS, Pavia, Italia
[3] Sorbonne Université, IRD, CNRS, MNHN, Laboratoire d'Océanographie et du Climat: Expérimentations et Approches Numériques, LOCEAN, Paris, France
[4] Laboratoire des Sciences du Climat et de l'Environnement (LSCE), CEA, CNRS, UVSQ, Gif-sur-Yvette, France
[5] Université Félix Houphouët Boigny, LAPAMF-UFR SSMT, Abidjan, Côte d'Ivoire
[6] ESPACE-DEV, Montpellier, France

[*]Contact: marco.gaetani@iusspavia.it

Supplementary Material

Table S1. Precipitation metric indices in West Africa (listed in the first column): in the 2nd column, 1986-2005 multi-model mean and spread (in brackets); in the 3rd column, multi-model absolute and relative (in brackets) change in 2080-2099; in the 4th and 5th columns, the fraction of the model simulating a significant positive and negative change, respectively. JD stands for Julian Day.

	1986-2005 mean	2080-2099 difference	Positive change	Negative change
West Sahel cumulated precipitation [mm/year]	571 (530-629)	-75 (-13%)	24%	69%
East Sahel cumulated precipitation [mm/year]	383 (353-441)	+135 (+35%)	69%	10%
West Sahel Wet Days [day]	56.2 (54.1-58.6)	-11.0 (-20%)	3%	72%
East Sahel Very Wet Days [day]	2.5 (2.1-3.5)	+2.6 (+103%)	72%	7%
Rainy Season Onset Date [JD]	126 (121-133)	+9	72%	10%
Rainy Season Retreat Date [JD]	286 (281-292)	+5	52%	3%
Rainy Season Length [day]	144 (133-150)	-4 (-3%)	14%	48%

Table S2. Time of emergence (TOE) assessment for July-to-September cumulated precipitation in West and East Sahel, number of wet days in West Sahel and number of very wet days in East Sahel, onset date, retreat date and length of the rainy season in West Africa. For each method, multi-model agreement, TOE and confidence interval (CI) are reported. TOE for positive/negative/no trend is highlighted in red/blue/green. Bold font indicates robust agreement (larger than 2/3 of the ensemble).

	KS test			smoothing			linear trend		
	agreement	TOE	CI	agreement	TOE	CI	agreement	TOE	CI
West Sahel cumulated precipitation	62%	2041	24	59%	2058	42	62%	2047	25
East Sahel cumulated precipitation	**72%**	**2017**	**34**	66%	2027	39	59%	2040	28
West Sahel Wet Days	**69%**	**2018**	**26**	**76%**	**2027**	**41**	**76%**	**2019**	**21**
East Sahel Very Wet Days	**83%**	**2023**	**38**	**76%**	**2033**	**50**	**72%**	**2043**	**25**
Rainy Season Onset Date	**79%**	**2042**	**57**				52%	NO	
Rainy Season Retreat Date	55%	2039	40						
Rainy season Length	52%	NO					62%	NO	

Table S3. Time of emergence (TOE) assessment for July-to-September cumulated precipitation in West and East Sahel, number of wet days in West Sahel and number of very wet days in East Sahel, using different time windows for the 'KS test' method: 15, 21 and 25 years, respectively. For each method, multi-model agreement, TOE and confidence interval (CI) are reported. TOE for positive/negative/no trend is highlighted in red/blue/green. Bold font indicates robust agreement (larger than 2/3 of the ensemble).

	**KS test (15-year)**			**KS test (21-year)**			**KS test (25-year)**		
	**Agreement**	**TOE**	**CI**	**agreement**	**TOE**	**CI**	**agreement**	**TOE**	**CI**
**West Sahel cumulated precipitation**	62%	2051	27	62%	2041	24	66%	2040	20
**East Sahel cumulated precipitation**	**69%**	**2022**	**47**	**72%**	**2017**	**34**	66%	2018	1
**West Sahel Wet Days**	**72%**	**2023**	**35**	**69%**	**2018**	**26**	**76%**	**2019**	**30**
**East Sahel Very Wet Days**	**79%**	**2027**	**43**	**83%**	**2023**	**38**	**76%**	**2018**	**10**
**Rainy Season Onset Date**	66%	2036	46	**80%**	**2042**	**57**	**79%**	**2038**	**42**
**Rainy Season Retreat Date**	52%	2047	43	55%	2039	40	55%	2039	39
**Rainy season Length**	52%	NO		52%	NO		52	NO	

Table S4. List of CMIP5 models analyzed, including details on resolution and modelling centers.

**Modelling Centre**	**Model**	**Resolution (lon×lat×level)**
Commonwealth Scientific and Industrial Research Organization (CSIRO) and Bureau of Meteorology (BOM), Australia	ACCESS1-0	1.25°×1.875°×38
	ACCESS1-3	
Beijing Climate Center, China Meteorological Administration	bcc-csm1-1	1.875°×1.875°×16
	bcc-csm1-1-m	
College of Global Change and Earth System Science, Beijing Normal University	BNU-ESM	2.81°×2.81°×26
Canadian Centre for Climate Modelling and Analysis	CanESM2	2.790°×2.81°×35
Centro Euro-Mediterraneo per I Cambiamenti Climatici	CMCC-CESM	3.443°×3.75°×39
	CMCC-CM	0.748°×0.75°×31
	CMCC-CMS	3.711°×3.75°×95
Centre National de Recherches Météorologiques/Centre Européen de Recherche et Formation Avancée en Calcul Scientifique	CNRM-CM5	1.4°×1.4°×31
Commonwealth Scientific and Industrial Research Organization in collaboration with Queens land Climate Change Centre of Excellence	CSIRO-Mk3-6-0	1.875°×1.875°×18
NOAA Geophysical Fluid Dynamics Laboratory	GFDL-CM3	2°×2.5°×48
	GFDL-ESM2G	2°×2.5°×24
	GFDL-ESM2M	
Met Office Hadley Centre (additional HadGEM2-ES realizations contributed by Instituto Nacional de Pesquisas Espaciais)	HadGEM2-AO	1.25°×1.875°×38
	HadGEM2-CC	
	HadGEM2-ES	
Institute for Numerical Mathematics	Inmcm4	1.5°×2°×21
Institut Pierre-Simon Laplace	IPSL-CM5A-LR	1.9°×3.75°×39
	IPSL-CM5A-MR	1.25°×2.5°×39
	IPSL-CM5B-LR	1.9°×3.75°×39
Atmosphere and Ocean Research Institute (University of Tokyo), National Institute for Environmental Studies, and Japan Agency for Marine-Earth Science and Technology	MIROC5	1.4°×1.4°×40
Japan Agency for Marine-Earth Science and Technology, Atmosphere and Ocean Research Institute (University of Tokyo), and National Institute for Environmental Studies	MIROC-ESM	2.8125°×2.8125°×80
	MIROC-ESM-CHEM	
Max-Planck-Institut für Meteorologie (Max Planck Institute for Meteorology)	MPI-ESM-LR	1.8653°×1.875°×47
	MPI-ESM-MR	1.8653°×1.875°×95
Meteorological Research Institute	MRI-CGCM3	1.12148°×1.125°×48
	MRI-ESM1	
Norwegian Climate Centre	NorESM1-M	1.9°×2.5°×26

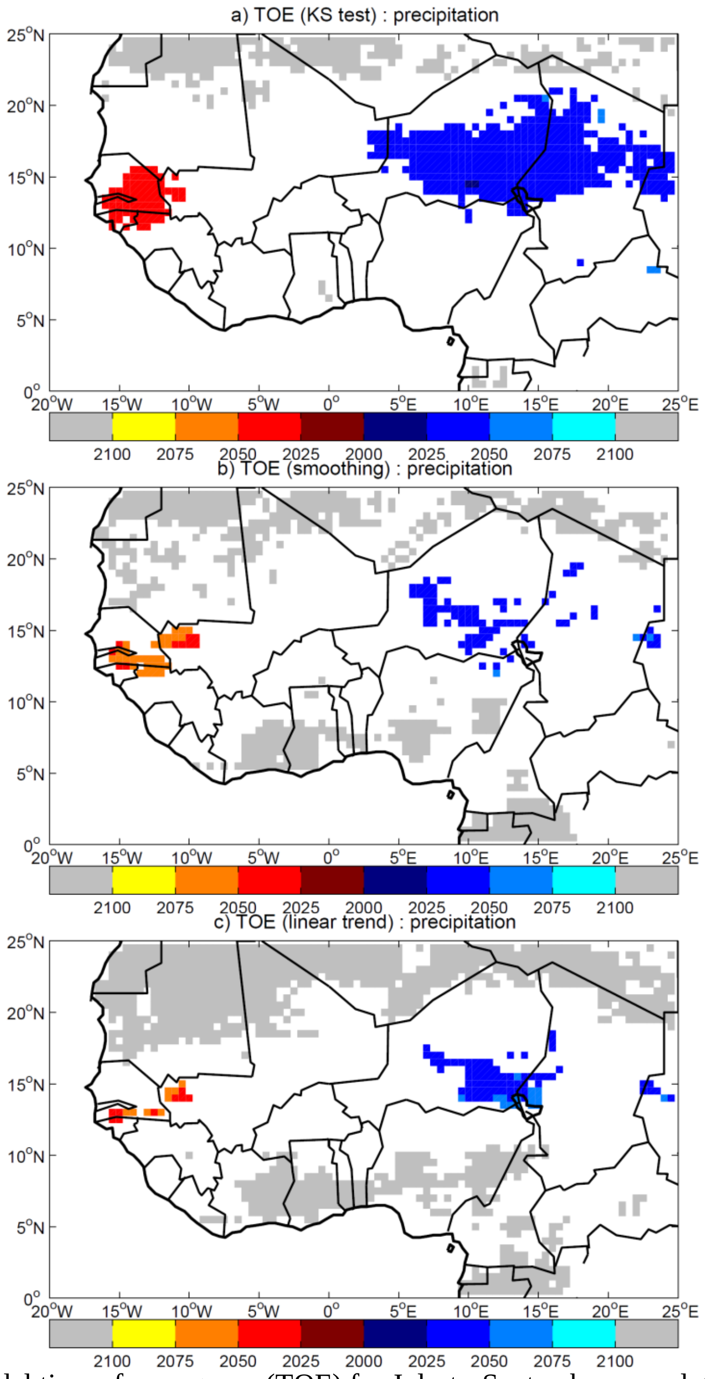

Figure S1. Multi-model time of emergence (TOE) for July-to-September cumulated precipitation, estimated by using (a) 'KS test', (b) 'smoothing' and (c) 'linear trend' methods. Blue/red/grey shadings display TOE for positive/negative/no trend in cumulated precipitation, based on 2/3 multi-model agreement. White areas indicate that multi-model TOE cannot be assessed (see Section 2 for details on the assessment of multi-model TOE).

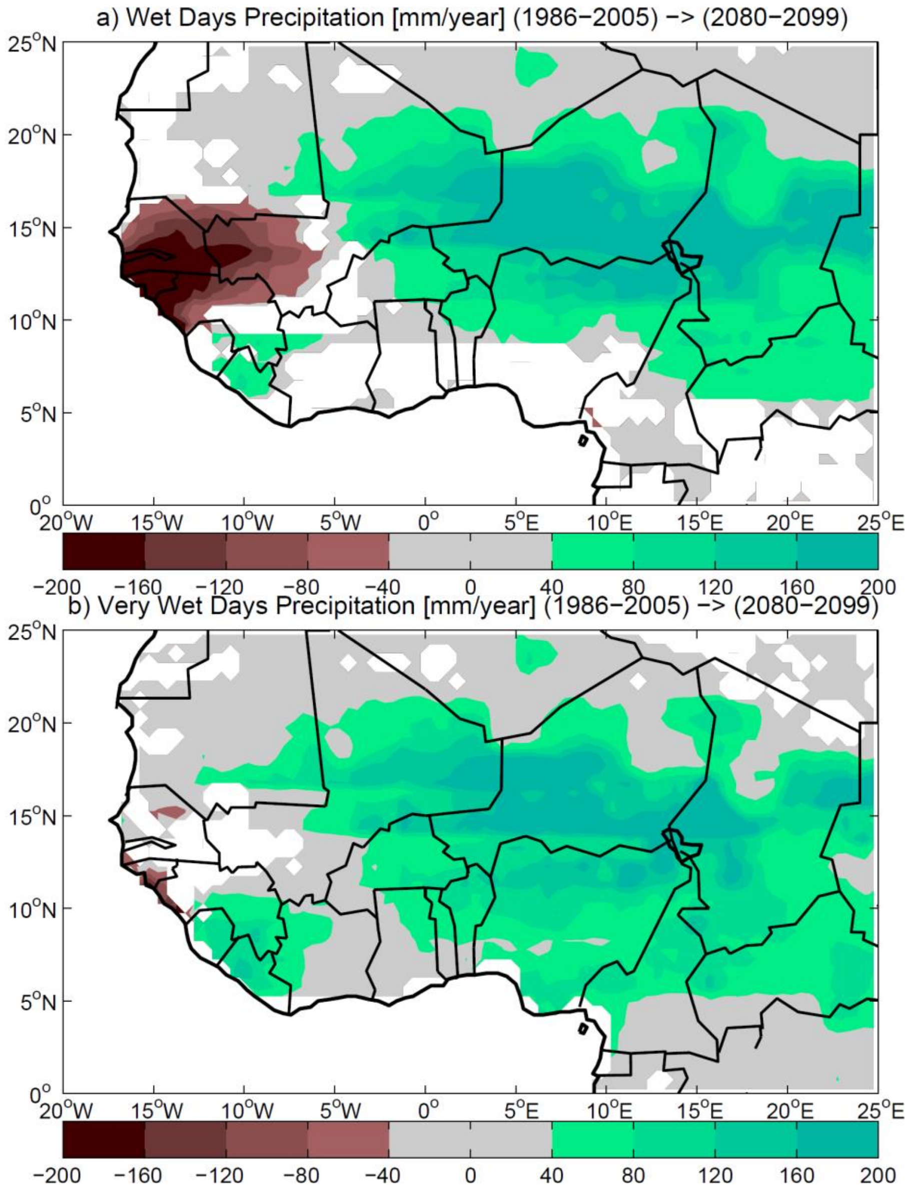

Figure S2. Ensemble mean change in July-to-September cumulated precipitation [mm/year] associated with (a) wet and (b) very wet days during the 21st century, computed as the difference between 2080-2099 and 1986-2005 averages. Significant values are displayed, after significance is assessed with a Student’s t-test at 95% confidence level.

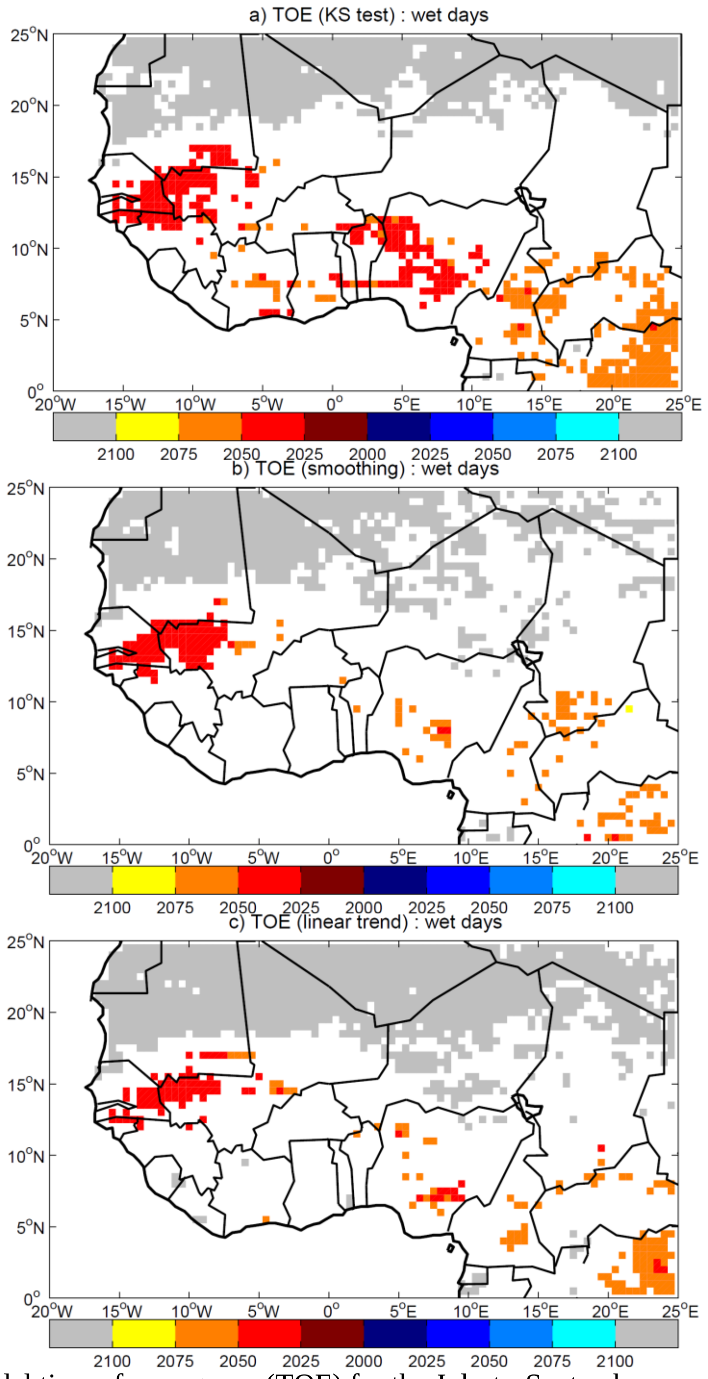

Figure S3. Multi-model time of emergence (TOE) for the July-to-September number of wet days, estimated by using (a) ‘KS test’, (b) ‘smoothing’ and (c) ‘linear trend’ methods. Blue/red/grey shadings display TOE for positive/negative/no trend in the number of wet days, based on 2/3 multi-model agreement. White areas indicate that multi-model TOE cannot be assessed (see Section 2 for details on the assessment of multi-model TOE).

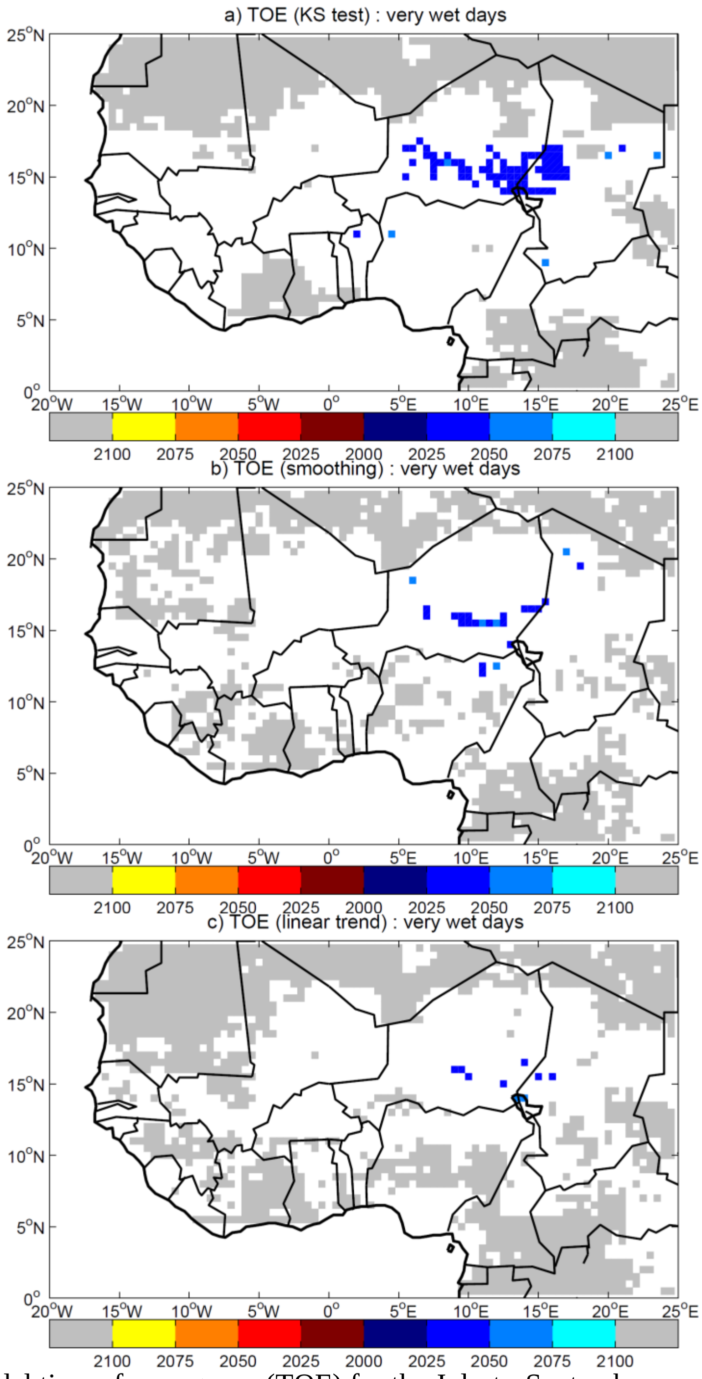

Figure S4. Multi-model time of emergence (TOE) for the July-to-September number of very wet days, estimated by using (a) ‘KS test’, (b) ‘smoothing’ and (c) ‘linear trend’ methods. Blue/red/grey shadings display TOE for positive/negative/no trend in the number of very wet days, based on 2/3 multi-model agreement. White areas indicate that multi-model TOE cannot be assessed (see Section 2 for details on the assessment of multi-model TOE).

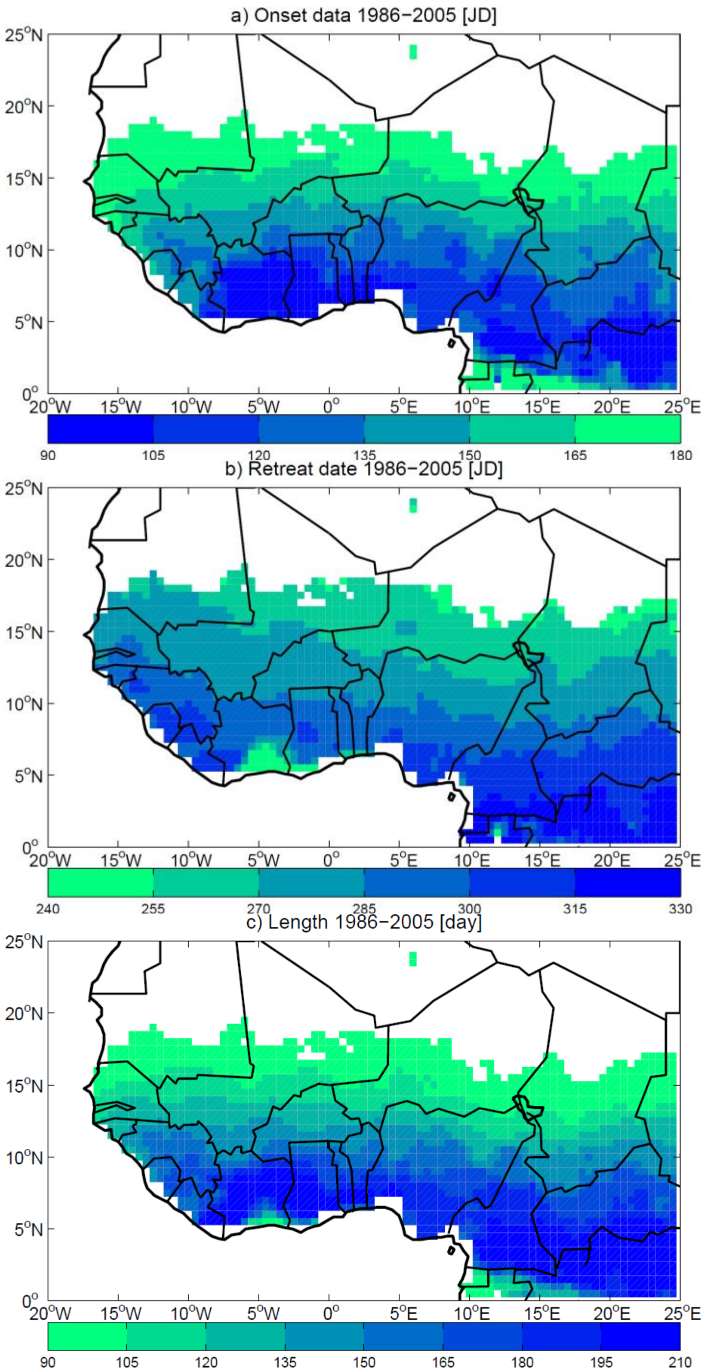

Figure S5. Ensemble mean of (a) onset and (b) retreat date and (c) length of the monsoonal season at the end of 20th century (1986-2005 average). JD stands for Julian Day.

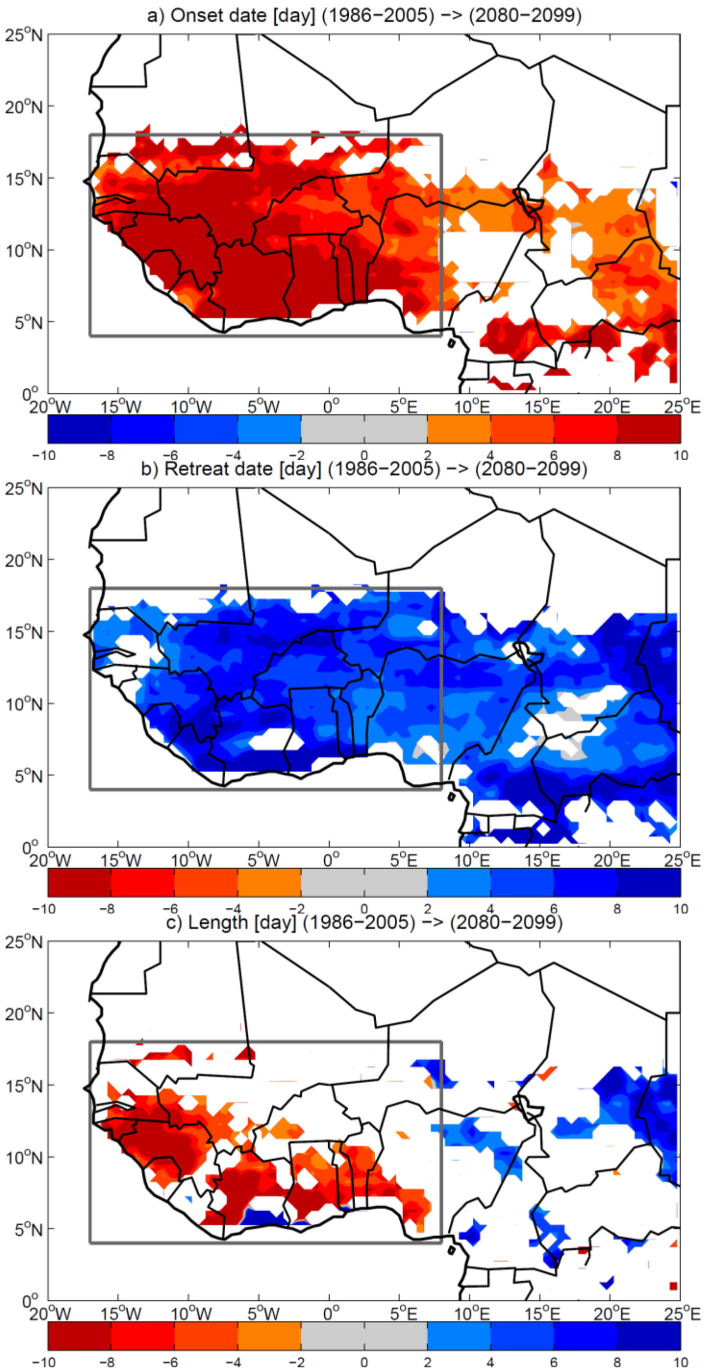

Figure S6. Ensemble mean change in (a) onset and (b) retreat date and (c) length of the monsoonal season during the 21st century, computed as the difference between 2080-2099 and 1986-2005 averages. Significant values are displayed, after significance is assessed with a Student's t-test at 95% confidence level; the grey rectangle shows the domain where the West Africa index is computed.

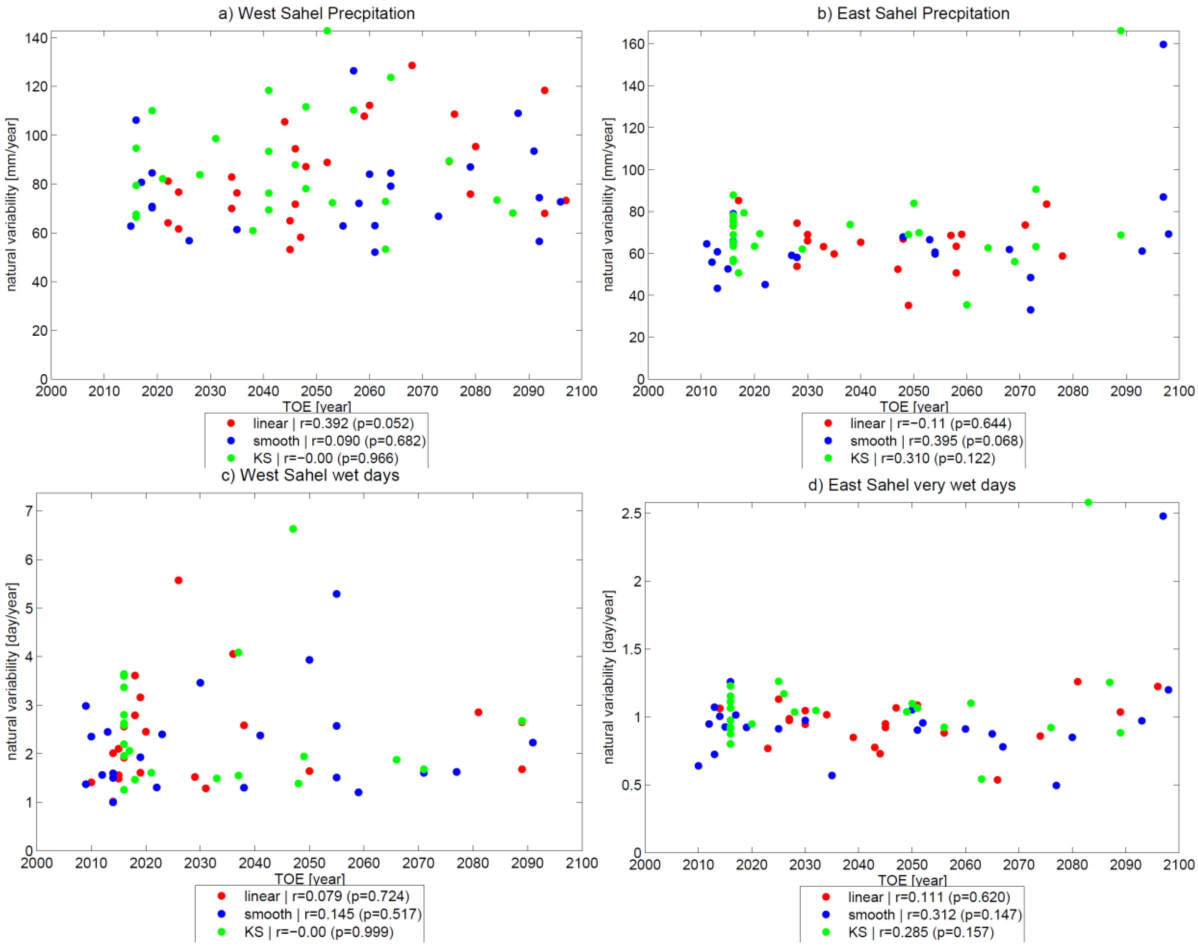

Figure S7. Precipitation metric indices for individual models: scatter plot of time of emergence (TOE) vs natural variability for July-to-September cumulated precipitation in (a) West and (b) East Sahel, (c) number of wet days in West Sahel and (d) number of very wet days in East Sahel. Natural variability is defined in 'linear trend' and 'smoothing' as the residual standard deviation in the period 1950-2005, and in 'KS test' as the standard deviation of the raw time series. In the boxes, correlation coefficients and p-values are displayed.

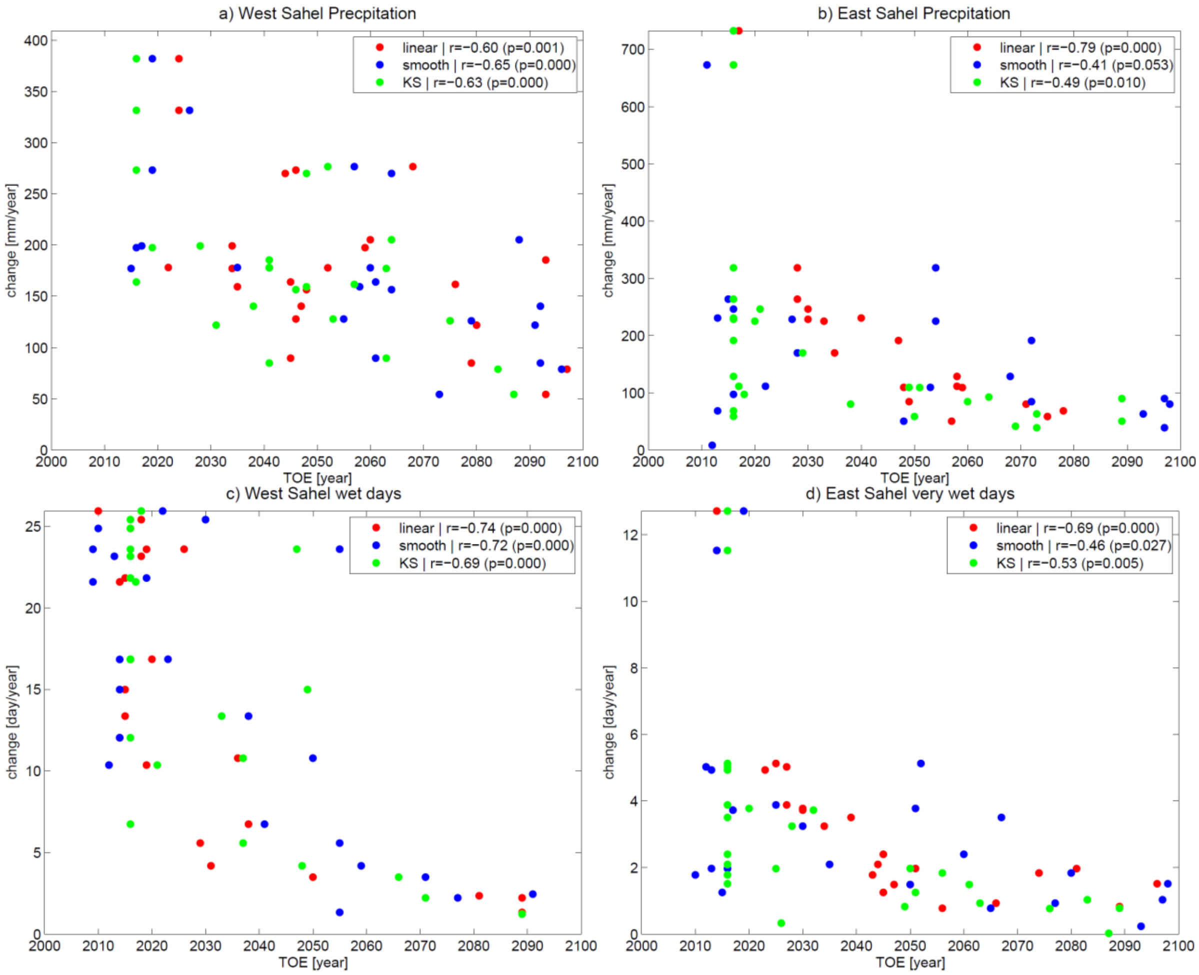

Figure S8. Precipitation metric indices for individual models: scatter plot of time of emergence (TOE) vs end-of-century changes defined as the difference between 2080-2099 and 1986-2005 averages for July-to-September cumulated precipitation in (a) West and (b) East Sahel, (c) number of wet days in West Sahel and (d) number of very wet days in East Sahel. In the boxes, correlation coefficients and p-values are displayed. When computed for log values, anti-correlations increase for cumulated precipitation in West Sahel (from -0.74 to -0.90 for 'linear trend', from -0.72 to -0.79, for 'smoothing' and from -0.69 to -0.82 for 'KS test', respectively) and the number of very wet days in East Sahel (from -0.69 to -0.79 for 'linear trend', from -0.46 to -0.59 for 'smoothing' and from -0.53 to -0.69 for 'KS test', respectively).

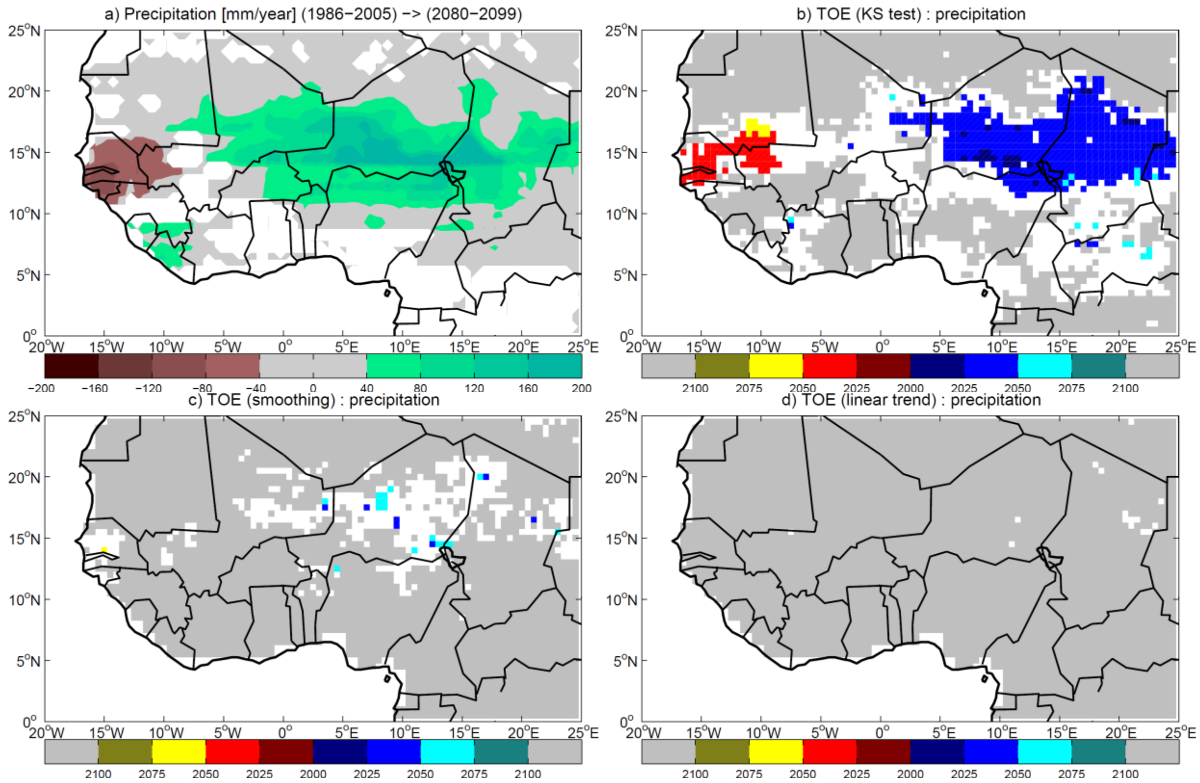

Figure S9. (a) Ensemble mean change in July-to-September cumulated precipitation in the *rcp45* scenario, computed as the difference between 2080-2099 and 1986-2005 averages. Significant values are displayed, after significance is assessed with a Student's t-test at 95% confidence level. Multi-model time of emergence (TOE) for cumulated precipitation, estimated by using (b) 'KS test', (c) 'smoothing' and (d) 'linear trend' methods. Blue/red/grey shadings display TOE for positive/negative/no trend in cumulated precipitation, based on 50% multi-model agreement. White areas indicate that multi-model TOE cannot be assessed (see Methods for details on the assessment of multi-model TOE).

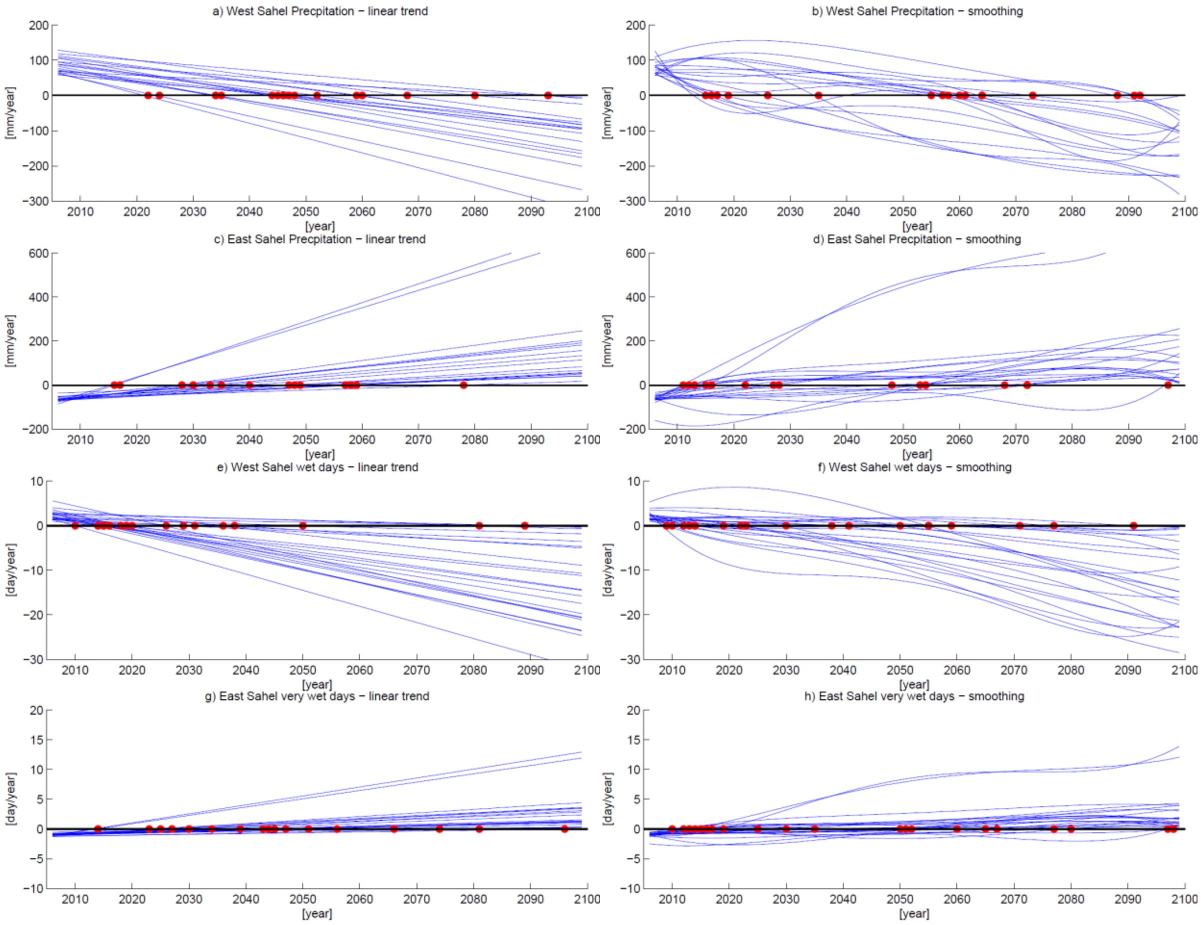

Figure S10. Climate change signal computed as linear trend (left panels) and 4th-degree polynomial smoothing (right panels), for cumulated precipitation in (a, b) West and (c, d) East Sahel, (e, f) the number of wet days in West Sahel and (g, h) the number of very wet days in East Sahel. Time series are normalized at year 2006 and at the natural variability threshold (see Section 2 for details). Red bullets indicate the crossing of the threshold, i.e. the time of emergence (TOE) of climate change. Only time series for models within the subset with the highest multi-model agreement, i.e. models used to compute the multi-model TOE, are displayed.
